# Bioelectrical Impedance Analysis—An Easy Tool for Quantifying Body Composition in Infancy?

**DOI:** 10.3390/nu12040920

**Published:** 2020-03-27

**Authors:** Jaz Lyons-Reid, Leigh C. Ward, Timothy Kenealy, Wayne Cutfield

**Affiliations:** 1 Liggins Institute, The University of Auckland, Auckland 1023, New Zealand; j.lyons-reid@auckland.ac.nz; 2 School of Chemistry and Molecular Biosciences, The University of Queensland, St. Lucia, Brisbane, QLD 4072, Australia; l.ward@uq.edu.au; 3 Department of Medicine and Department of General Practice and Primary Health Care, The University of Auckland, Auckland 1023, New Zealand; t.kenealy@auckland.ac.nz; 4 Liggins Insitute and A Better Start – National Science Challenge, The University of Auckland, Auckland 1023, New Zealand

**Keywords:** bioelectrical impedance analysis, infant, body composition

## Abstract

There has been increasing interest in understanding body composition in early life and factors that may influence its evolution. While several technologies exist to measure body composition in infancy, the equipment is typically large, and thus not readily portable, is expensive, and requires a qualified operator. Bioelectrical impedance analysis shows promise as an inexpensive, portable, and easy to use tool. Despite the technique being widely used to assess body composition for over 35 years, it has been seldom used in infancy. This may be related to the evolving nature of the fat-free mass compartment during this period. Nonetheless, a number of factors have been identified that may influence bioelectrical impedance measurements, which, when controlled for, may result in more accurate measurements. Despite this, questions remain in infants regarding the optimal size and placement of electrodes, the standardization of normal hydration, and the influence of body position on the distribution of water throughout the body. The technology requires further evaluation before being considered as a suitable tool to assess body composition in infancy.

## 1. Body composition in infancy

In 1858, Albert von Bezold first chemically analysed a stillborn infant [1]. Since then, there has been great interest in understanding how an infant’s body composition evolves as it grows. While a healthy infant is born with 10–15% body fat, this percentage doubles by six months of age, and slowly decreases thereafter until adiposity rebounds, typically around age six years [2,3,4]. However, this is highly variable, with a number of early life factors influencing adiposity [5,6,7,8,9,10,11]. In addition to changes in fat mass during infancy, total body water as a percentage of body weight decreases from approximately 80% to 60% across the first year of life. This decrease in total body water is accompanied by changes in the proportion of intra- and extracellular water, alongside increases in osseous mineral [2,3]; meaning that while adults maintain a constant hydration of fat-free mass (73%) [12], this varies during infancy. Reference data from Fomon [2] and Butte [3] suggest that the hydration of fat-free mass ranges between 83% and 79% in the first year of life. This is important, as techniques that assess body composition in infancy rely on reference data to inform prediction rather than directly measure body composition.

## 2. Moving Away from a Two-Compartment Model

**Figure 1:**
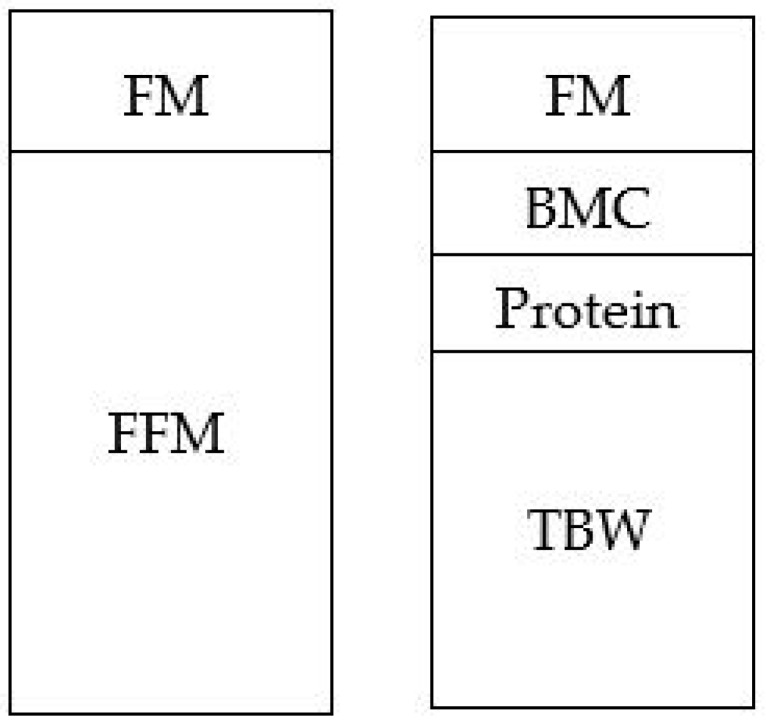
Components of a two-compartment and a four-compartment model of body composition. Abbreviations: FM, fat mass; FFM, fat-free mass; BMC, bone mineral content; TBW, total body water.

While von Bezold directly analysed body composition using cadaveric chemical analysis, it goes without saying that the destructive nature of this technique prohibits its use in standard practice. Instead, clinicians and researchers must rely on indirect measures. These techniques typically divide the body into two notional compartments—fat mass and fat-free mass (Figure 1) [13]—while assuming that the composition of the fat-free mass compartment is constant. The fat-free mass compartment contains total body protein, osseous and non-osseous mineral, intra- and extracellular water, and marginal amounts of essential lipid and glycogen. As these components are known to change as an infant ages, information from reference infants is used in predicting body composition. However, this process is imperfect. The calculations assume that the reference data is both “true” and relevant to all infants. Given the number of assumptions that were involved in developing the references [14], and the variable nature of body composition, for example, based on ethnicity [15,16,17], one must question the validity of techniques which rely on these reference values and associated assumptions.

A four-component model is considered the “gold-standard” technique for assessing body composition in infancy (Figure 1) [18]. A multi-component model removes some uncertainty around the composition of the fat-free mass compartment in an individual. This is done by measuring total body water by isotope dilution, bone mineral content by dual-energy X-ray absorptiometry, and body density by air displacement plethysmography. This information is then input into a formula such as Lohman’s to determine body fat percentage [19]. The technique is time- and resource-intensive, and requires a great deal of cooperation from the infant. Thus, the technique is seldom used [3].

## 3. Bioelectrical Impedance Analysis—An Inexpensive, Portable and Easy Tool

Bioelectrical impedance analysis is a technique that measures the opposition (impedance) to a harmless alternating electrical current as it passes through the body’s water pool typically between upper to lower limbs. The technique has long been used to assess body water, and in 1985 Lukaski et al. [20] first used bioimpedance to assess fat-free mass in healthy adults. Since then, there has been an exponential increase in the number of publications per year related to the technology [21]. Despite its increase in popularity, bioelectrical impedance remains infrequently used in infancy, with techniques such as air displacement plethysmography (i.e., the PEA POD Infant Body Composition System) predominating. This is perhaps due to the need to develop population-specific prediction equations and the purported poor accuracy of the technique. However, technological advancements mean that impedance can now be measured over a broad range of frequencies, with bioelectrical impedance spectroscopy offering advantages over single- and multiple-frequency bioimpedance devices. Bioelectrical impedance spectroscopy can better distinguish between the impedance of intra- and extracellular fluid compartments [22,23]. Furthermore, the use of these impedances in combination with Cole modelling and Hanai mixture theory means that population-specific predictors are not required [24,25]. However, it is unclear how the resistivity coefficients used in these modelling equations change as an infant grows [26], therefore the inclusion of population-specific adjustments is thought to increase accuracy at the individual level [27]. Despite this, to date, bioelectrical impedance spectroscopy has seldom been used to assess body composition in infancy [28,29,30,31]. Indeed, recent studies frequently rely on older previously published single-frequency bioimpedance predictive equations, for example those by Kushner et al. [32], to inform prediction of body composition among their participants [33,34].

Collins et al. [28] developed resistivity coefficients for use in Hanai mixture theory from 99 preterm infants at three weeks of age using deuterium and bromide dilution as reference standards. While the predicted values from bioelectrical impedance spectroscopy correlated well with those from the criterion methods, when internally validated there were small biases but large limits of agreement (total body water (TBW): −0.6% (36% to −37%); extracellular fluid (ECF): −4.0% (32% to −39%)), suggesting the technique may not be suitable at the individual level in this age group.

Lingwood and colleagues [29] were the first to use bioimpedance spectroscopy to assess fat-free mass in infancy. The authors developed predictive equations using the PEA POD as a reference standard among 77, 54, 55, and 53 infants at birth, six weeks, three months, and 4.5 months of age, respectively. Overall, impedance added little to the predictive power of the equations, however, predictive power improved with increasing age. Indeed, the inclusion of impedance values showed improvements in prediction beyond standard anthropometry only in those infants aged over three months.

Tint et al. [30] developed predictive equations from 173 and 140 Singaporean neonates at birth and at two weeks of age, respectively. The authors found minimal correlation between fat-free mass and impedance at birth (*R* = −0.204, *p* = 0.007), however, this improved in the older infants (*R* = −0.438, *p* < 0.001). When cross-validated with Lingwood et al.’s [29] cohort, the predictive equations performed similarly only when the infants were age-matched. Likewise, Gridneva et al. [31] found that the performance of bioelectrical impedance equations was strongly influenced by whether there was an appropriate age-match for their population of infants. It should be noted that both Tint et al. [30] and Gridneva et al. [31] used resistance at 50 kHz, as is standard with single frequency bioimpedance devices. Lingwood et al. [29], however, found better prediction of fat-free mass when using other impedance values, for example, impedance at the characteristic frequency (Zc) and resistance at zero frequency (R_0_). Thus, bioelectrical impedance spectroscopy remains largely unexplored for measuring fat-free mass in infancy.

The aforementioned techniques of single- and multi-frequency bioimpedance analysis, although widely used, have been reviewed extensively elsewhere [18,26]. Likewise, bioelectrical impedance vector analysis (BIVA), a technique which involves plotting bioimpedance parameters against a reference ellipse, but does not allow for the absolute quatification of body composition, is beyond the scope of this paper. We direct the reader to Toffano et al.’s [35] article.

## 4. Limitations of Bioelectrical Impedance Technologies

### 4.1. Is it Actually That Easy?

Recently, the relevant literature was reviewed and a general lack of standardization, or reporting of such standardization, was noted in articles related to bioelectrical impedance analysis (BIA) [36]. Rather, several articles include broad statements akin to “duplicate measurements were taken with subjects lying in the supine position with electrodes placed on the left side of the body on the hands and feet”. This is unsurprising, as the technique’s ease of use may give the user a false sense of confidence, and without adequate understanding of the technology, there may be an under-appreciation of the long list of factors that may influence measurements [37]. While formal guidelines exist for use in adults, currently none exist for use in paediatric populations [38,39].

### 4.2. A Call for Standardization

In recognition of a lack of standardization of methodology Brantlov and colleagues [37] reviewed the literature and identified a number of critical factors that are known to, or may, influence bioimpedance measurements in children. The authors called on international societies to develop consensus guidelines for standardization but recommended the review be used in planning future studies in the interim. Table 1 summarises these factors.

While the review included literature relevant to all paediatric populations, special considerations apply during infancy. Questions remain regarding the impact and feasibility of several of the guidelines in a population known for their poor compliance and great inter-individual variation. These include:

1. What is the optimal size and placement of electrodes?
2. Can “normal” hydration be standardized?
3. How does time spent supine influence water distribution within the body?

#### 4.2.1. Electrodes

While adult guidelines dictate that electrodes of at least 4 cm^2^ be placed at least 5 cm apart, on the hands and feet (if doing whole-body analysis), in infants this is not feasible due to their small size. Some authors have addressed this by cutting the electrodes in half [28,40,41,42], contrary to manufacturer’s recommendations [43,44]. The impact of electrode modification has not yet been elucidated, however, one manufacturer claims that the larger surface area is required to obtain an accurate reading in dual and multi-frequency devices [44], although Svensson et al. [45] found that using half-size electrodes made less than 1% difference to measured resistance. In contrast, several groups have investigated whether placement of the sense and source electrodes within 5 cm of each other influences impedance measurements in infants. The National Institutes of Health guidelines state that a 1 cm displacement of electrodes can result in a 2% change in resistance [38]. While some support this notion and have found that impedance values are significantly impacted by inter-electrode distance [41,46], Sesmero et al. [47] found that while inter-electrode distance impacted on resistance at high frequencies, there was little consequence at low frequencies. This is significant as others have found that resistance at R_0_ has the best predictive ability for determining body composition in infancy, likely related to the greater variability in impedance which is observed at the high frequency end [29]. In addition to the effects of inter-electrode distance, the intrinsic impedance of electrodes may influence impedance measurements [45,48]. While guidelines support the use of device-specific electrodes [39], research suggests that electrodes can be substituted so long as there is not an electrode mismatch [45,49,50,51,52].

#### 4.2.2. Hydration

It is well established that hydration influences bioimpedance measurements, however, standardizing factors that influence hydration may prove challenging in infants. Compounding this, hydration is highly variable during this period, complicating the establishment of normal hydration. While in adults it is recommended that bioelectrical impedance analysis be conducted in a fasted state [38,39], this is neither feasible nor ethical in infancy. Sesmero et al. [47] demonstrated that time after consumption of milk influenced impedance values, with no effect of milk volume. Although overall the effect of fasting was minimal, this varied based on the age of the infant. Gridneva et al. [53] found that while changes in impedance values were apparent between pre- and post-feed measurements, these were not statistically significant, and this remained true across infants of all ages (2, 5, 9 and 12 months). Thus, it is unclear whether feeding may influence measurements in infancy, and given these conflicting results, more research is required to establish the magnitude of the effects of fasting versus feeding. In addition, it is recommended that individuals void prior to measurement as this has been related to a 1.0% error in measurements in adults [54]. However, while standardizing fasting in infancy is challenging, standardizing voiding is near impossible. Though standardization of the factors influencing hydration may not be feasible, improved reporting and further research may lead to a better understanding of the significance of these factors.

#### 4.2.3. Movement

In addition to being in a hydrated state, bioelectrical impedance analysis guidelines in adults dictate that subjects should obstain from exercise for several hours prior to measurement [38,39]. Although infants are relatively sedentary, accelerometry data has recently highlighted that infants are increasingly active as they age [55]. Currently, no research has explored the influence of infant physical activity on impedance measurements, however, effects are likely mitigated by time spent supine. In adults, guidelines dictate that subjects should be supine for 4–10 min prior to assessment [38,39] as time spent supine can influence impedance values [56]. It takes approximately five min for fluid stabilisation to occur to allow measurement of total body water [57], and extended periods are required in order to establish extra- and intracellular fluid stabilisation [57,58]. Of course, this is not always feasible with infants, and the effects of body position need to be elucidated, particularly given the greater variability in water distribution that is observed in this population. Subjects are also required to be relaxed during measurement, as movement artefact has been found to affect impedance values in infants, with increased movement translating to increased resistance [47]. While movement artefact may be limited by wrapping the infant in a blanket, Sesmero and colleagues [47] found swaddling increased resistance values compared to when the infant was unrestrained. Of course, this approach requires great care to ensure correct abduction of the limbs is maintained as skin-to-skin contact has been associated with error ranging from 18% to 43% in adults [56]. Although not always feasible, an alternative approach for limiting movement during measurement is to take the measurement while the infant is asleep. Fortunately, while achieving optimal measurements may be challenging, those known to be confounded by movement can easily be discarded.

### 4.3. Validating against a TRUE Criterion Method

While bioelectrical impedance analysis is often criticised for poor accuracy, Ward [21] noted that no technique is free of error, and researchers may be expecting performance beyond the limits of a device which is simply measuring the resistance to a current. Indeed, the small bias and large limits of agreement often seen with bioimpedance are not dissimilar to the techniques it is validated against, for example, the PEA POD [29,59]. Given that body composition is so variable during infancy, multi-component models are considered “gold-standard” and should be used to validate new, simpler technologies [18]. Despite this, bioelectrical impedance analysis has not been validated against a multi-component model in infants, nor have several of the tools it is commonly validated against. For example, while dual-energy X-ray absorptiometry has been validated against a four-component model in adults [60], adolescents [61,62,63,64], and children [61,62], this has not been carried out in infants. Likewise, although the PEA POD has been validated against a four-component model [59], this was completed using fat-free mass density data from Butte’s reference. Despite this, several studies using the PEA POD have relied on density data from Fomon’s reference [29,65,66,67,68,69,70]. This may, in part, be due to research that has found better alignment with isotope dilution when using Fomon’s reference compared to Butte’s [65,67,71].

## 5. Concluding Remarks

In a climate that is increasingly focused on the development of fat mass and evolving obesity, there is a need for an inexpensive, portable, and easy to use tool to assess body composition in infancy. Bioelectrical impedance analysis, notably spectroscopy, offers promise; however, to do the technology justice we need to move away from blindly accepting the numbers presented on the screen. We must work towards establishing clear standardized methodologies and reporting criteria, and accept the limitations of the technology. Future research should investigate the critical factors identified by Brantlov and colleagues [37], and groups using the technology in populations of infants should aim to standardize these factors where possible. Validation against true criterion methods is needed together with guidelines for standardized use in infancy.

## Figures and Tables

**Table 1:** Critical factors of bioelectrical impedance analysis in children: are they achievable in infancy? Modified from Brantlov et al. [37].

*Critical Factors*	Recommendations	Achievable?
*Choice of BIA device*	Use the same device consistently throughout the study.	Y
*Anthropometry*	Weight and height should be measured at ±0.1 kg and ±0.5 cm, respectively, and measured at the time of the BIA test.	Y
	Weight should be measured naked.	Y
	Length should be measured with an infant measuring board, measuring mat or measuring rod.	Y
	Self-reported measurements should not be used.	Y
*Fasting*	Perform measurement after a fast of at least 4 h.	N
*Hydration*	Subjects should be normally hydrated.	N
*Voiding*	Voiding should be done prior to measurement.	N
*Exercise*	Intense physical activity should be limited for a minimum of four hours prior to measurement.	N
*Clothing*	Ensure that no metals are in the clothing.	Y
*Electrodes*	Use BIA electrodes supplied by the manufacturer.	Y
	Use electrodes with a surface area ≥4 cm^2^.	N
*Skin preparation*	Clean with alcohol before placement of the electrodes.	Y
*Positioning of electrodes*	Place at the dorsal surfaces of the wrist and ankle.	Y
	Apply voltage electrodes at the midline between the prominent bone ends of the wrist and the ankle.	Y
	Place current electrodes 5 cm distal to these positions.	N
	Specify on which side of the body measurements are made.	Y
*Body position*	Subjects should be supine for at least 4 – 10 min before measurements are taken.	N
*Movement*	Subjects should be relaxed during measurements.	N
*Electrical interference*	Arms and legs should be abducted within a 30–45° angle from the trunk.	Y
	Measurements should be made on non-conductive surfaces.	Y
	Ensure that the device cables are not touching the ground, subjects, metal objects, routed near high voltage equipment, are used in a neutral environment and are not intertwined.	Y
*Temperature*	Measurements should be taken at an ambient temperature.	Y
*Time of measurement*	For longitudinal follow-up studies, measurements should be performed at the same time of day.	Y
*Calibration*	Calibrate BIA device regularly.	Y
*Operator*	Ensure proper training in order to get valid and reproducible measurements.	Y
*Data quality*	Test of data quality should be performed by inspection of impedance values in BIA devices, and by inspection of Cole plots in BIS devices.	Y
*Number of measurements*	Measurements should be continued until stable values are achieved, measured to the nearest Ohm.	Y
	The average of a minimum of three repeated measurements should be calculated.	Y

Abbreviations: Y, yes; N, no; BIA, bioelectrical impedance analysis; BIS, bioelectrical impedance spectroscopy.

## References

[1] von Bezold A. (1858). Das chemische Skelett der Wirbelthiere. Z. Fur Wiss. Zool..

[2] Fomon S.J., Haschke F., Ziegler E.E., Nelson S.E. (1982). Body composition of reference children from birth to age 10 years. Am. J. Clin. Nutr..

[3] Butte N.F., Hopkinson J.M., Wong W.W., Smith E.O.B., Ellis K.J. (2000). Body Composition during the First 2 Years of Life: An Updated Reference. Pediatric Res..

[4] Rolland-Cachera M.F., Deheeger M., Bellisle F., Sempé M., Guilloud-Bataille M., Patois E. (1984). Adiposity rebound in children: A simple indicator for predicting obesity. Am. J. Clin. Nutr..

[5] Magalhães E.I.d.S., Lima N.P., Menezes A.M.B., Gonçalves H., Wehrmeister F.C., Assunção M.C.F., Horta B.L. (2019). Maternal smoking during pregnancy and offspring body composition in adulthood: Results from two birth cohort studies. BMJ Open.

[6] Landhuis C.E., Poulton R., Welch D., Hancox R.J. (2008). Childhood sleep time and long-term risk for obesity: A 32-year prospective birth cohort study. Pediatrics.

[7] Lowe W.L., Scholtens D.M., Lowe L.P., Kuang A., Nodzenski M., Talbot O., Catalano P.M., Linder B., Brickman W.J., Clayton P. (2018). Association of Gestational Diabetes With Maternal Disorders of Glucose Metabolism and Childhood Adiposity. JAMA.

[8] Rito A.I., Buoncristiano M., Spinelli A., Salanave B., Kunešová M., Hejgaard T., García Solano M., Fijałkowska A., Sturua L., Hyska J. (2019). Association between Characteristics at Birth, Breastfeeding and Obesity in 22 Countries: The WHO European Childhood Obesity Surveillance Initiative—COSI 2015/2017. Obes. Facts.

[9] Taveras E.M., Rifas-Shiman S.L., Sherry B., Oken E., Haines J., Kleinman K., Rich-Edwards J.W., Gillman M.W. (2011). Crossing growth percentiles in infancy and risk of obesity in childhood. Arch. Pediatric Adolesc. Med..

[10] Voerman E., Santos S., Patro Golab B., Amiano P., Ballester F., Barros H., Bergström A., Charles M.A., Chatzi L., Chevrier C. (2019). Maternal body mass index, gestational weight gain, and the risk of overweight and obesity across childhood: An individual participant data meta-analysis. PLoS Med..

[11] Whitaker R.C., Pepe M.S., Wright J.A., Seidel K.D., Dietz W.H. (1998). Early adiposity rebound and the risk of adult obesity. Pediatrics.

[12] Wang Z., Deurenberg P., Wang W., Pietrobelli A., Baumgartner R.N., Heymsfield S.B. (1999). Hydration of fat-free body mass: Review and critique of a classic body-composition constant. Am. J. Clin. Nutr..

[13] Weber D.R., Leonard M.B., Zemel B.S. (2012). Body composition analysis in the pediatric population. Pediatric Endocrinol. Rev..

[14] Fomon S.J., Nelson S.E. (2002). Body composition of the male and female reference infants. Annu. Rev. Nutr..

[15] Gallagher D., Heymsfield S.B., Heo M., Jebb S.A., Murgatryod P.R., Sakamoto Y. (2000). Healthy percentage body fat ranges: An approach for developing guidelines based on body mass index. Am. J. Clin. Nutr..

[16] Nightingale C.M., Rudnicka A.R., Owen C.G., Cook D.G., Whincup P.H. (2011). Patterns of body size and adiposity among UK children of South Asian, black African-Caribbean and white European origin: Child Heart And health Study in England (CHASE Study). Int. J. Epidemiol..

[17] Yajnik C.S., Fall C.H.D., Coyaji K.J., Hirve S.S., Rao S., Barker D.J.P., Joglekar C., Kellingray S. (2003). Neonatal anthropometry: The thin-fat Indian baby. The Pune Maternal Nutrition Study. Int. J. Obes..

[18] Demerath E.W., Fields D.A. (2014). Body composition assessment in the infant. Am. J. Hum. Biol..

[19] Lohman T.G. (1989). Assessment of body composition in children. Pediatric Exerc. Sci..

[20] Lukaski H.C., Johnson P.E., Bolonchuk W.W., Lykken G.I. (1985). Assessment of fat-free mass using bioelectrical impedance measurements of the human body. Am. J. Clin. Nutr..

[21] Ward L.C. (2019). Bioelectrical impedance analysis for body composition assessment: Reflections on accuracy, clinical utility, and standardisation. Eur J. Clin. Nutr..

[22] Cornish B.H., Thomas B.J., Ward L.C. (1993). Improved prediction of extracellular and total body water using impedance loci generated by multiple frequency bioelectrical impedance analysis. Phys. Med. Biol..

[23] De Lorenzo A., Andreoli A., Matthie J., Withers P. (1997). Predicting body cell mass with bioimpedance by using theoretical methods: A technological review. J. Appl. Physiol. (1985).

[24] Matthie J.R. (2008). Bioimpedance measurements of human body composition: Critical analysis and outlook. Expert Rev. Med. Devices.

[25] Cole K.S., Cole R.H. (1941). Dispersion and Absorption in Dielectrics I. Alternating Current Characteristics. J. Chem. Phys..

[26] Lingwood B.E. (2013). Bioelectrical impedance analysis for assessment of fluid status and body composition in neonates--the good, the bad and the unknown. Eur. J. Clin. Nutr..

[27] Kyle U.G., Bosaeus I., De Lorenzo A.D., Deurenberg P., Elia M., Gómez J.M., Heitmann B.L., Kent-Smith L., Melchior J.C., Pirlich M. (2004). Bioelectrical impedance analysis—Part I: Review of principles and methods. Clin. Nutr..

[28] Collins C.T., Reid J., Makrides M., Lingwood B.E., McPhee A.J., Morris S.A., Gibson R.A., Ward L.C. (2013). Prediction of body water compartments in preterm infants by bioelectrical impedance spectroscopy. Eur. J. Clin. Nutr..

[29] Lingwood B.E., Storm van Leeuwen A.M., Carberry A.E., Fitzgerald E.C., Callaway L.K., Colditz P.B., Ward L.C. (2012). Prediction of fat-free mass and percentage of body fat in neonates using bioelectrical impedance analysis and anthropometric measures: Validation against the PEA POD. Br. J. Nutr..

[30] Tint M.-T., Ward L.C., Soh S.E., Aris I.M., Chinnadurai A., Saw S.M., Gluckman P.D., Godfrey K.M., Chong Y.-S., Kramer M.S. (2016). Estimation of fat-free mass in Asian neonates using bioelectrical impedance analysis. Br. J. Nutr..

[31] Gridneva Z., Hepworth A.R., Ward L.C., Lai C.T., Hartmann P.E., Geddes D.T. (2017). Determinants of body composition in breastfed infants using bioimpedance spectroscopy and ultrasound skinfolds-methods comparison. Pediatric Res..

[32] Kushner R.F., Schoeller D.A., Fjeld C.R., Danford L. (1992). Is the impedance index (ht2/R) significant in predicting total body water?. Am. J. Clin. Nutr..

[33] Wood K., Mantzioris E., Lingwood B., Couper J., Makrides M., Gibson R.A., Muhlhausler B.S. (2018). The effect of maternal DHA supplementation on body fat mass in children at 7 years: Follow-up of the DOMInO randomized controlled trial. Prostaglandins Leukot. Essent. Fat. Acids.

[34] Wall C.R., Hill R.J., Lovell A.L., Matsuyama M., Milne T., Grant C.C., Jiang Y., Chen R.X., Wouldes T.A., Davies P.S.W. (2019). A multicenter, double-blind, randomized, placebo-controlled trial to evaluate the effect of consuming Growing Up Milk “Lite” on body composition in children aged 12-23 mo. Am. J. Clin. Nutr..

[35] Toffano R.B.D., Hillesheim E., Margutti A.V.B., Camelo Junior J.S., Ferraz I.S., Del Ciampo L.A., Monteiro J.P. (2018). Bioelectrical Impedance Vector Analysis in Healthy Term Infants in the First Three Months of Life in Brazil. J. Am. Coll. Nutr..

[36] Brantlov S., Jodal L., Lange A., Rittig S., Ward L.C. (2017). Standardisation of bioelectrical impedance analysis for the estimation of body composition in healthy paediatric populations: A systematic review. J. Med. Eng. Technol..

[37] Brantlov S., Ward L.C., Jodal L., Rittig S., Lange A. (2017). Critical factors and their impact on bioelectrical impedance analysis in children: A review. J. Med. Eng. Technol..

[38] National Institutes of Health (1996). Bioelectrical impedance analysis in body composition measurement: National Institutes of Health Technology Assessment Conference Statement. Am. J. Clin. Nutr..

[39] Kyle U.G., Bosaeus I., De Lorenzo A.D., Deurenberg P., Elia M., Manuel Gómez J., Lilienthal Heitmann B., Kent-Smith L., Melchior J.C., Pirlich M. (2004). Bioelectrical impedance analysis-part II: Utilization in clinical practice. Clin. Nutr..

[40] Piccoli A., Fanos V., Peruzzi L., Schena S., Pizzini C., Borgione S., Bertino E., Chiaffoni G., Coppo R., Tatò L. (2002). Reference values of the bioelectrical impedance vector in neonates in the first week after birth. Nutrition.

[41] Raghavan C.V., Super D.M., Chatburn R.L., Savin S.M., Fanaroff A.A., Kalhan S.C. (1998). Estimation of total body water in very-low-birth-weight infants by using anthropometry with and without bioelectrical impedance and H2[(18)O]. Am. J. Clin. Nutr..

[42] Tang W., Ridout D., Modi N. (1997). Assessment of total body water using bioelectrical impedance analysis in neonates receiving intensive care. Arch. Dis. Child. -Fetal Neonatal Ed..

[43] ImpediMed SFB7 Brochure. https://www.impedimed.com/wp-content/products/SFB7/SFB7_CA_Brochure.pdf.

[44] Bodystat FAQ’S. https://www.bodystat.com/support/.

[45] Svensson B.J., Dylke E.S., Ward L.C., Kilbreath S.L. (2019). Electrode Equivalence for Use in Bioimpedance Spectroscopy Assessment of Lymphedema. Lymphat. Res. Biol..

[46] Sidhu J.S., Triggs E.J., Charles B.G., Tudehope D.I. (1994). Electrode placement in neonatal bioelectrical impedance analysis. Med. Biol. Eng. Comput..

[47] Sesmero M.A., Mazariegos M., Pedrón C., Jones J., Solomons N.W. (2005). Bioimpedance electrical spectroscopy in the first six months of life: Some methodologic considerations. Nutrition.

[48] Nescolarde L., Lukaski H., De Lorenzo A., de-Mateo-Silleras B., Redondo-Del-Rio M.P., Camina-Martin M.A. (2016). Different displacement of bioimpedance vector due to Ag/AgCl electrode effect. Eur. J. Clin. Nutr..

[49] Bogónez-Franco P., Nescolarde L., Bragós R., Rosell-Ferrer J., Yandiola I. (2009). Measurement errors in multifrequency bioelectrical impedance analyzers with and without impedance electrode mismatch. Physiol. Meas..

[50] Buendía R., Bogónez-Franco P., Nescolarde L., Seoane F. (2012). Influence of electrode mismatch on Cole parameter estimation from Total Right Side Electrical Bioimpedance Spectroscopy measurements. Med. Eng. Phys..

[51] Caicedo-Eraso J.C., González-Correa C.H., González-Correa C.A. (2012). Use of electrocardiogram (ECG) electrodes for Bioelectrical Impedance Analysis (BIA). J. Phys. Conf. Ser..

[52] González-Correa C.H., Caicedo-Eraso J.C. (2018). Looking for optimum ECG electrodes for bioelectrical impedance analysis (BIA). The need for evaluation. Nutr. Hosp..

[53] Gridneva Z., Hepworth A.R., Ward L.C., Lai C.T., Hartmann P.E., Geddes D.T. (2016). Bioimpedance spectroscopy in the infant: Effect of milk intake and extracellular fluid reservoirs on resistance measurements in term breastfed infants. Eur. J. Clin. Nutr..

[54] Gonzalez C., Evans J.A., Smye S.W., Holland P. (1999). Variables affecting BIA measurement of body water. Med. Biol. Eng. Comput..

[55] Benjamin-Neelon S.E., Bai J., Ostbye T., Neelon B., Pate R.R., Crainiceanu C. (2020). Physical Activity and Adiposity in a Racially Diverse Cohort of US Infants. Obesity.

[56] Kushner R.F., Gudivaka R., Schoeller D.A. (1996). Clinical characteristics influencing bioelectrical impedance analysis measurements. Am. J. Clin. Nutr..

[57] Gibson A., Beam J., Alencar M., Zuhl M., Mermier C. (2014). Time course of supine and standing shifts in total body, intracellular and extracellular water for a sample of healthy adults. Eur. J. Clin. Nutr..

[58] Segal K.R., Van Loan M., Fitzgerald P.I., Hodgdon J.A., Van Itallie T.B. (1988). Lean body mass estimation by bioelectrical impedance analysis: A four-site cross-validation study. Am. J. Clin. Nutr..

[59] Ellis K.J., Yao M., Shypailo R.J., Urlando A., Wong W.W., Heird W.C. (2007). Body-composition assessment in infancy: Air-displacement plethysmography compared with a reference 4-compartment model. Am. J. Clin. Nutr..

[60] Van Der Ploeg G.E., Withers R.T., Laforgia J. (2003). Percent body fat via DEXA: Comparison with a four-compartment model. J. Appl. Physiol. (1985).

[61] Sopher A.B., Thornton J.C., Wang J., Pierson R.N., Heymsfield S.B., Horlick M. (2004). Measurement of percentage of body fat in 411 children and adolescents: A comparison of dual-energy X-ray absorptiometry with a four-compartment model. Pediatrics.

[62] Wells J.C.K., Haroun D., Williams J.E., Wilson C., Darch T., Viner R.M., Eaton S., Fewtrell M.S. (2010). Evaluation of DXA against the four-component model of body composition in obese children and adolescents aged 5-21 years. Int. J. Obes..

[63] Wong W.W., Hergenroeder A.C., Stuff J.E., Butte N.F., Smith E.O., Ellis K.J. (2002). Evaluating body fat in girls and female adolescents: Advantages and disadvantages of dual-energy X-ray absorptiometry. Am. J. Clin. Nutr..

[64] Gately P.J., Radley D., Cooke C.B., Carroll S., Oldroyd B., Truscott J.G., Coward W.A., Wright A. (2003). Comparison of body composition methods in overweight and obese children. J. Appl. Physiol. (1985).

[65] Andersen G.S., Girma T., Wells J.C., Kæstel P., Leventi M., Hother A.-L., Michaelsen K.F., Friis H. (2013). Body composition from birth to 6 mo of age in Ethiopian infants: Reference data obtained by air-displacement plethysmography. Am. J. Clin. Nutr..

[66] Carberry A.E., Colditz P.B., Lingwood B.E. (2010). Body Composition From Birth to 4.5 Months in Infants Born to Non-Obese Women. Pediatric Res..

[67] Eriksson B., Löf M., Forsum E. (2010). Body composition in full-term healthy infants measured with air displacement plethysmography at 1 and 12 weeks of age. Acta Paediatr..

[68] Fields D.A., Gilchrist J.M., Catalano P.M., Giannì M.L., Roggero P.M., Mosca F. (2011). Longitudinal body composition data in exclusively breast-fed infants: A multicenter study. Obesity.

[69] Henriksson H., Eriksson B., Forsum E., Flinke E., Henriksson P., Löf M. (2017). Longitudinal assessment of body composition in healthy Swedish children from 1 week until 4 years of age. Eur. J. Clin. Nutr..

[70] Roggero P., Giannì M.L., Orsi A., Piemontese P., Amato O., Liotto N., Morlacchi L., Taroni F., Fields D.A., Catalano P.M. (2010). Quality of Growth in Exclusively Breast-Fed Infants in the First Six Months of Life: An Italian Study. Pediatric Res..

[71] Eriksson B., Löf M., Eriksson O., Hannestad U., Forsum E. (2011). Fat-free mass hydration in newborns: Assessment and implications for body composition studies. Acta Paediatr..

